# Infections submicroscopiques à *Plasmodium* spp. chez les patients fébriles au Togo

**DOI:** 10.48327/mtsi.v5i1.2025.553

**Published:** 2025-02-27

**Authors:** Diwaba Carmel TEOU, Essoham ATABA, Smaila ALIDOU, Kossi YAKPA, Efoe SOSSOU, Manani HEMOU, Agueregna ABDOU-KERIM, Awèréou KOTOSSO, Lidaw Déassoua BAWE, Didier MÉNARD, Ameyo Monique DORKENOO

**Affiliations:** 1 Faculté des sciences, Université de Lomé, Boulevard Eyadema, 01BP 1515 Lomé, Togo; 2 Programme national de lutte contre le paludisme, ministère de la Santé, de l'hygiène publique et de l'accès universel aux soins, Quartier administratif, 01BP 518 Lomé, Togo; 3 Département de santé publique, Unité de formation et de recherche en sciences de la santé, Université Joseph KI-ZERBO, Ouagadougou, Burkina Faso; 4 Service des laboratoires, Centre hospitalier universitaire Sylvanus Olympio, 198 rue de l’Hôpital, Tokoin Hôpital, BP 57, Lomé, Togo; 5 Département de pédiatrie, Campus hospitalier universitaire de Lomé, Boulevard Gnassingbé Eyadéma, Campus, Cité OUA - 03 BP 30284 Lomé, Togo; 6 Institut national d'hygiène, ministère de la Santé et de l'hygiène publique, Quartier administratif, 01BP 1396 Lomé, Togo; 7 Service des maladies infectieuses et tropicales, CHU Sylvanus Olympio, Lomé, Togo; 8 Centre hospitalier des armées de Lomé, Togo; 9 Malaria Genetics and Resistance Team (MEGATEAM), UR 3073 - Pathogens Host Arthropods Vectors Interactions, Université de Strasbourg, F-67000 Strasbourg, France; Laboratory of Parasitology and Medical Mycology, CHU de Strasbourg, 67000 Strasbourg, France; Malaria Parasite Biology and Vaccines, Institut Pasteur, Université Paris Cité, 75015 Paris, France; Institut universitaire de France, Paris, France; 10 Faculté des Sciences de la santé, Université de Lomé, Boulevard Eyadema, 01BP 1515 Lomé, Togo

**Keywords:** Prévalence, Infections submicroscopiques, Paludisme, *Plasmodium falciparum*, Réservoir, Élimination, Togo, Afrique subsaharienne, Prevalence, Submicroscopic infections, Malaria, *Plasmodium falciparum*, Reservoir, Elimination, Togo, Sub-Saharan Africa

## Abstract

**Objectif:**

Les infections submicroscopiques à *Plasmodium*, la plupart du temps non détectées par les techniques de diagnostic de routine, constituent un réservoir potentiel qui participe au maintien de la transmission du paludisme dans la population. Pour atteindre l'objectif d’élimination fixé par l’Organisation mondiale de la santé, il est donc crucial d'identifier tous les porteurs de parasites et de les traiter efficacement avec les antipaludiques recommandés. L'objectif de cette étude était d'estimer la proportion des infections submicroscopiques à *Plasmodium* spp., non détectées par la microscopie, chez les patients symptomatiques suspects de paludisme consultant dans les centres de santé au Togo et d'identifier les facteurs qui y sont associés.

**Matériel et méthodes:**

Une étude transversale a été conduite entre septembre 2021 et janvier 2022 et entre juillet et décembre 2022 dans trois formations sanitaires du Togo. Chaque patient suspect de paludisme a bénéficié d'un prélèvement de sang capillaire pour la recherche des espèces plasmodiales par goutte épaisse/frottis sanguin (GE/FS) et PCR. Un modèle de régression logistique a été utilisé pour évaluer les facteurs associés aux résultats parasitologiques.

**Résultats:**

Un total de 553 participants dont 44,6 % de sexe féminin avec un âge médian de 25 ans (± 2 ans) a été retenu. La proportion des infections à *Plasmodium* spp. détectées par GE/FS était de 25 % et par PCR de 29,1 %. La fréquence des infections submicroscopiques à *Plasmodium* spp. repérées par PCR chez les patients présentant une GE/FS négative à la microscopie était de 5,5 % (23/415) [IC 95 % : 3,7-8,2] et *P. falciparum* représentait l'espèce la plus fréquente (83 %, 19/23, IC 95 % : 60-94). Les participants des sites d'Anié et de Kouvé étaient plus fréquemment porteurs d'infections submicroscopiques.

**Conclusion:**

Cette étude fournit les données préliminaires de la fréquence des infections submicroscopiques à *Plasmodium* au Togo.

## Abstract (en)

**Objective:**

Submicroscopic *Plasmodium* infections, mostly undetected by routine diagnostic techniques, represent a potential reservoir that contributes to the continued transmission of malaria in the community. To achieve the World Health Organization's goal of malaria elimination, it is therefore essential to identify all parasite carriers and treat them effectively with recommended antimalarial drugs. The aim of this study was to estimate the proportion of submicroscopic *Plasmodium* spp. infections not detected by microscopy in symptomatic patients with suspected malaria attending health centers in Togo, and to identify the factors associated with it.

**Materials and methods:**

A cross-sectional study was conducted between September 2021 and January 2022 and between July and December 2022 in three health facilities in Togo. Each patient suspected of having malaria had a capillary blood sample taken for detection of *Plasmodium* species by thick blood film/blood smear (TBF/BS) and PCR. A logistic regression model was used to evaluate the factors linked to the parasitic results.

**Results:**

A total of 553 participants were selected, 44.6% of whom were female, with a median age of 25 years (± 2 years). The proportion of *Plasmodium* spp. infections detected by TBF/BS was 25% and by PCR 29.1%. The frequency of submicroscopic *Plasmodium* spp. infections detected by PCR in patients with negative microscopic TBF/BS was 5.5% (23/415) [95% CI: 3.7-8.2], and *P. falciparum* was the most common species (83%, 19/23, 95% CI: 60-94). Participants from the Anié and Kouvé sites were more likely to have submicroscopic infections.

**Conclusion:**

This study provides preliminary data on the incidence of submicroscopic *Plasmodium* infections in Togo.

## Introduction

La capacité des systèmes de santé des pays d'endémie palustre à détecter les infections à *Plasmodium* spp. est essentielle pour la surveillance et le contrôle efficace de l'endémie [30]. L’Organisation mondiale de la santé (OMS) [19] recommande l'utilisation des combinaisons thérapeutiques à base d'artémisinine (CTA) pour le traitement des cas non compliqués à *P. falciparum* et de l'artésunate par voie intraveineuse ou intramusculaire pendant au moins 24 h, complétée par une CTA pour les cas de paludisme grave.

Au Togo, en plus de ces recommandations implémentées depuis 2005 [11], la mise en œuvre réussie d'autres stratégies de prévention, notamment la distribution de moustiquaires à imprégnation durable, le traitement préventif intermittent avec la sulfadoxine-pyriméthamine chez les femmes enceintes et la chimio prévention du paludisme saisonnier chez les enfants de 3 à 59 mois avec l'association sulfadoxine-pyriméthamine et amodiaquine pendant la saison de forte transmission palustre, de même que l'amélioration du diagnostic et du traitement des cas confirmés pourraient expliquer la baisse observée de la prévalence du paludisme chez les enfants âgés de 6-59 mois, passée de 36 % à 28 % entre 2014 et 2017 [15]. S'agissant des décès, la létalité due au paludisme en 2022 était de 2,1 % contre 3,6% en 2019 [16]. Les directives nationales recommandent que tous les cas suspects de paludisme soient confirmés par une goutte épaisse (GE), un frottis sanguin (FS) ou un test de diagnostic rapide (TDR) avant tout traitement antipaludique. Du fait de leur facilité d'utilisation et de la mise à disposition rapide des résultats, les TDR ont été tout d'abord utilisés dans les formations sanitaires sans microscopie et dans la communauté par les agents de santé communautaire, puis récemment même dans les structures sanitaires avec microscopie. Les TDR actuellement utilisés sont spécifiques à l'espèce *P. falciparum* [16]. Toutefois, l'examen microscopique par GE et FS demeure la référence; il permet la mise en évidence des differents stades d'espèces plasmodiales et l'estimation de la densité parasitaire (DP). Le seuil de détection des espèces plasmodiales par cette approche, estimé entre 10 et 20 parasites/µl [9], reste bien plus élevé que celui de la biologie moléculaire (Polymerase Chain Reaction, PCR) estimé à 0,002 parasites/µl [29], permettant de déceler les cas d'infections submicroscopiques (IS). Dans les pays à ressources limitées et endémiques au paludisme, la PCR est souvent utilisée pour la confirmation biologique lors des études épidémiologiques et d’évaluation de l'efficacité des antipaludiques mais pas en routine [9, 12].

La proportion des IS varie selon le contexte épidémiologique et les populations atteintes [21, 27]; la contribution de ce réservoir au maintien de la transmission du paludisme dépend de l'endémicité et du taux de positivité des cas de paludisme détectés dans une zone donnée [12]. En effet, Okell *et al.* ont rapporté qu'au niveau mondial, la prévalence des IS est inversement corrélée à la DP [17]. D'autres études ont également suggéré que, en zone de faible transmission, la proportion d’IS est souvent plus forte que celles détectables par la microscopie et qu'elle risque d'augmenter au fur et à mesure que les programmes de lutte continueront à réduire l'intensité de la transmission du paludisme [5, 13]. En outre, il a été montré que la fréquence des IS est non négligeable dans les zones à forte transmission, et que ces infections joueraient un rôle dans le développement de l'immunité partielle non stérilisante contre le paludisme [26].

En Afrique de l’Ouest, des cas d’IS ont été documentés dans certaines études. Au Bénin, Agohossou *et al.* [2], ainsi que Telfils *et al.* [28] ont rapporté dans leurs études une prévalence d'infections submicroscopiques de 30,6 % et 30,4 %, respectivement. Au Ghana, Amoah *et al*. [4] ont observé que cette prévalence était de 33,8 %. Au Togo, les infections submicroscopiques n'ont pas encore été documentées. Cette étude a été menée en vue d'estimer la proportion des infections submicroscopiques à *Plasmodium* spp. non détectées à la microscopie chez les patients symptomatiques suspects de paludisme consultant dans les centres de santé au Togo et d'identifier les facteurs qui y sont associés.

## Matériel et méthodes

Une étude transversale a été conduite entre septembre 2021 et janvier 2022 à l'hôpital « La Providence » de Kouvé et à l'hôpital de district (HD) d’Anié, et de juillet à décembre 2022 au Centre hospitalier régional Lomé Commune (CHR-LC). L'hôpital « La Providence » de Kouvé, situé dans la région maritime, est à environ 76 km de Lomé et l’HD d’Anié, situé dans la région des plateaux, est à 188 km de Lomé. Ces deux formations sanitaires font partie des six sites sentinelles institués par le ministère de la Santé pour le suivi de l'efficacité des antipaludiques. Ils représentent le premier niveau de contact avec les patients. Par ailleurs, afin de prendre en compte le deuxième niveau de l'organisation pyramidale du système sanitaire du Togo, le CHR-Lomé commune, situé dans la région sanitaire du Grand-Lomé, structure de niveau intermédiaire, a été ajouté. Il couvre l'aire géographique de la capitale Lomé et ses banlieues. Ces trois sites représentent les trois régions sanitaires au sud du pays.

La population d’étude était constituée de patients symptomatiques, suspects de paludisme, pour lesquels des examens de GE/FS ont été prescrits. Tout patient, quel que soit son âge, reçu dans le service de consultation générale de l'un des établissements de santé participant à l’étude, présentant une température axillaire ≥ 37,5 °C (ou des antécédents de fièvre au cours des précédentes 24 heures avec suspicion de paludisme) était inclus dans l’étude. Il devait avoir donné son consentement éclairé (directement ou *via* les parents/tuteurs pour les enfants).

Après avoir complété le formulaire permettant de collecter les informations socio-démographiques, les signes cliniques présentés et les antécédents médicaux, chaque patient retenu a bénéficié d'un prélèvement de sang capillaire pour la réalisation de la GE/FS et de confettis sanguins sur du papier Wattman de type III.

La GE et le FS confectionnés sur la même lame étaient colorés au Giemsa 3 % après avoir fixé le FS au méthanol pendant quelques secondes [20]. Les lames après séchage étaient ensuite lues au microscope à l'objectif x 100 pour déterminer la positivité, identifier les espèces plasmodiales et pour estimer la DP. Cette DP était exprimée en nombre de plasmodies asexuées par microlitre de sang suivant la formule : nombre de plasmodies dénombrées x 8 000 / nombre de leucocytes dénombrés.

Pour extraire l’ADN, des confettis sanguins étaient découpés avec des ciseaux et transférés sur des plaques à 96 puits. L’ADN génomique a été extrait à l'aide du kit Qiagen RLT-plus^®^ selon le protocole du fabricant avec quelques modifications [1]. À chaque puits, ont été ajoutés 750 pl de tampon de lyse, 500 µL de tampon de lavage 1, 500 pl de tampon de lavage 2 et 100 pl de tampon d’élution (Tris-EDTA). L'extraction a été effectuée à la température et au temps d'incubation recommandés et à la vitesse de centrifugation indiquée par le fabricant. Enfin, les plaques d’élution contenant l’ADN extrait ont été stockées à -80 °C jusqu’à leur utilisation.

Les échantillons étaient criblés pour la présence d’ADN de *Plasmodium* spp. à l'aide d'un test PCR qualitatif en temps réel ciblant le gène cytochrome b *(screening).* Les produits pour les échantillons positifs ont ensuite été dilués au 1:10 et analysés à l'aide d'un test PCR en temps réel avec des amorces ciblant le même fragment de gène et spécifiques à chaque espèce (PCR pour les espèces *P. falciparum*, *P. vivax*, *P. malariae* et *P. ovale).* Tous les tests en temps réel ont été effectués à l'aide du *master mix PCR SYBR green* (Solis Biodyne, Tartu, Estonia) sur un extrait d’ADN de 5 µl avec le système de PCR en temps réel BioRad CFX96). Des détails sur le programme, la composition du mélange et les séquences d'amorces ont été décrits par Canier *et al.* [6, 7].

Les lames de GE/FS des sujets inclus étaient soumises à une relecture par deux microscopistes expérimentés [18]. Dans les cas où le coefficient de variation entre les résultats des deux microscopistes du contrôle de qualité était supérieur à 5 %, une troisième lecture a été faite par un autre microscopiste indépendant [20]. En cas de différence entre la DP des sites et celle de l’équipe de contrôle de qualité, nous avons considéré la DP du contrôle de qualité.

Pour chaque analyse par *PCR screening*, un ensemble de quatre contrôles (deux contrôles positifs dont un positif élevé et un faiblement positif, et deux contrôles négatifs correspondant à de l'eau) a été ajouté dans chaque plaque à 96 puits. Pour la validation des espèces, les constructions plasmidiques décrites précédemment ont été utilisées dans chaque test PCR [7].

Une formation des membres de l’équipe des sites et des superviseurs a été faite pour standardiser les méthodes de travail et garantir le bon déroulement de l'activité notamment pour le remplissage des questionnaires, la réalisation de la GE/FS et le prélèvement sur papier filtre.

La variable dépendante est une variable binaire indiquant la présence (positif : codé 1) ou l'absence (négatif : codé 0) d'une infection submicroscopique. Cette infection est définie comme une infection parasitaire de faible densité au stade sanguin du paludisme qui n'est pas détectée par la GE/FS, mais qui est uniquement détectée par la PCR, considérée comme la méthode de référence [18].

Les variables indépendantes incluent les sites d’études, à savoir le CHR-LC, l’HD d’Anié et l'hôpital Kouvé, ainsi que des caractéristiques sociodémographiques et cliniques. Le sexe des participants est distingué (féminin ou masculin) et l’âge réparti en trois groupes : les moins de 5 ans, ceux âgés de 5 à 34 ans et les patients de 35 ans et plus. La température est classée comme normale <37,5 °C ou élevée ≥37,5 °C et l'automédication codée comme absente (non) ou présente (oui). Concernant la profession, les participants sont catégorisés comme salariés, travailleurs à la tâche, élèves/étudiants, enfants et autres. Enfin, la résidence distingue les urbains des ruraux. Ces variables ont été sélectionnées sur la base de leur pertinence dans la littérature existante et ont fait l'objet d'analyses dans les études similaires [12, 14, 23, 32].

Toutes les données collectées ont été saisies en une base de données dans le tableur Microsoft Excel (Microsoft Corp, Redmond, Washington, USA) et analysées à l'aide du logiciel *stata* 16 (StataCorp LLC). Les données qualitatives ont été présentées en proportions suivies de leur IC95 % et celles quantitatives décrites en médianes avec leurs intervalles interquartiles. Les échantillons négatifs à la fois à la GE/FS et à la PCR ont été comparés à ceux négatifs à la GE/FS et positifs à la PCR. Pour ces analyses, le test exact de Fisher a été utilisé pour la comparaison des groupes et la régression logistique pour identifier une association possible avec les facteurs étudiés. Une valeur de p < 0,05 a été considérée comme statistiquement significative. Les variables candidates à la régression logistique multiple étaient celles ayant une valeur de p inférieure ou égale à 0,20 lors de l'analyse préliminaire.

Le protocole d’étude a obtenu l'accord du Comité de bioéthique pour la recherche en santé (CBRS) du Togo (n°021/2021/CBRS du 24 mai 2022). De plus, un consentement signé a été exigé des adultes ou des parents/tuteurs lorsqu'il s'agissait d'enfants. Tout patient détecté positif à la GE/FS a été adressé aux cliniciens des établissements participants pour la prise en charge gratuite avec de l'artéméther-luméfantrine ou la dihydroar-témisinine-pipéraquine comme recommandé par le Programme national de lutte contre le paludisme (PNLP).

## Résultats

**Figure 1:**
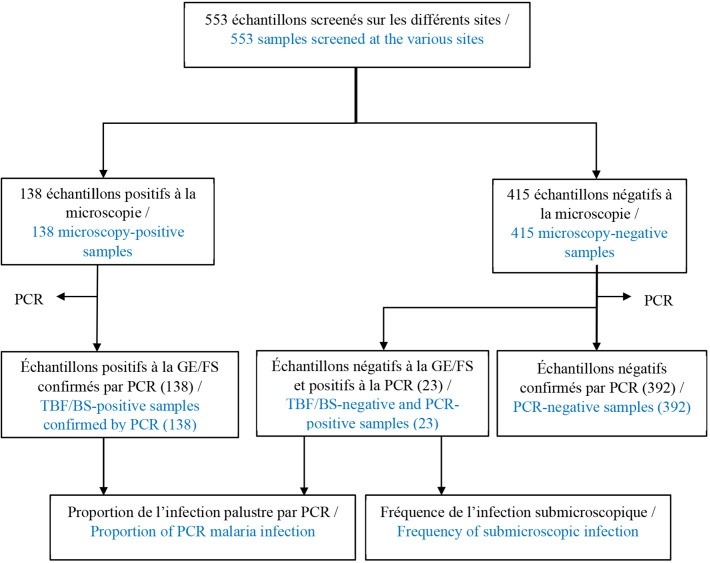
Diagramme de l'étude

Un total de 553 patients suspects de paludisme a été inclus sur l'ensemble des sites : 138 avaient une GE positive (Tableau I). La recherche des cas d'infections submicroscopiques par *PCR screening* a été effectuée sur les 415 prélèvements négatifs par la GE/FS (Fig. 1).

**Tableau I:** Nombre total d'échantillons inclus par site

Sites	Kouvé n (%)	HD Anié n (%)	CHR-LC n (%)
GE/FS			
négative (n = 415)	99 (17,9)	113 (20,4)	203 (36,7)
positive (n = 138)	35 (6,3)	103 (18,6)	0 (0)
total (n = 553)	134 (24,2)	216 (39,1)	203 (36,7)
PCR			
négative (n = 392)	89 (16,1)	103 (18,6)	200 (36,2)
positive (n = 161)	45 (8,1)	113 (20,4)	3 (0,5)
total (n = 553)	134 (24,2)	216 (39,1)	203 (36,7)

L’âge moyen des patients était de 25 ans (± 2 ans) et le sex-ratio (M/F) de 1,2. La température corporelle à l'admission ≥ 37,5 °C a été notée chez 77,8 % d'entre eux, les autres ayant eu un antécédent fébrile dans les 24 heures avant la consultation. Les données sociodémographiques des patients sont résumées dans le Tableau III.

La détection du génome plasmodial par PCR s'est faite sur l'ensemble des 553 inclus (Tableau I). En comparant les deux méthodes, la PCR a détecté davantage de cas positifs, soit 29,1 % (161/553) contre 25 % (138/553) en microscopie (Tableau I). La fréquence des infections submicroscopiques à *Plasmodium* spp. a été de 5,5 % (23/415) [IC 95 % : 3,7-8,2]. La majorité de ces infections était à *P. falciparum*, soit 83% (19/23) [IC 95 % : 60-94] des cas. *P. malariae* était trouvé dans 9 % (2/23) [IC 95 % : 2-31] de ces infections submicroscopiques, de même que l'association *P. falciparum- P. malariae*, dans 9 % (2/23) [IC 95 % : 2-31] des cas (Tableau II). Aucun cas d'infection submicroscopique à *P. vivax* ou *P. ovale* n'a été identifié parmi les patients étudiés.

**Tableau II:** Fréquence de la parasitémie submicroscopique et des espèces de *Plasmodium* identifiées

Sites	Kouvé	HD Anié	CHR-LC	Total [IC 95 %]
GE/FS négative	99	113	203	415
submicroscopie n (%)	10 (10,1)	10 (8,8)	3 (1,5)	23 (5,5) [03,7-08,2]
espèces plasmodiales				
Pf*, n (%)	9	7	3	19
Pm*, n (%)	1	1	0	2
*Pf/Pm,* n (%)	0	2	0	2

* *Pf : Plasmodium falciparum, Pm : Plasmodium malariae*

Parmi les 415 échantillons négatifs à la GE/FS et testés par PCR, les participants des sites d’Anié et de Kouvé avaient respectivement 6 (RC, 6,47 [IC 95 % : 1,74-24,03]; p = 0,005) et 7 (RC, 7,49 [IC 95 % : 2,01-27,88]; p = 0,003) fois plus de risque d'avoir une IS. Bien que la fréquence de ces infections soit plus élevée chez les moins de cinq ans, aucune différence significative en fonction de l’âge ni du sexe n'a été observée. L'automédication, la profession et la résidence n’étaient pas non plus associées à l'infection submicroscopique (Tableau III).

**Tableau III:** Association entre les caractéristiques démographiques et les infections submicroscopiques à *Plasmodium* spp

Caractéristiques	Fréquence n (%)	Infections submicroscopiques n (%)	Rapport de cotes (IC 95 %)	p-value
Sites d’étude				
CHR-LC	203 (48,9)	3(1,5)	1	
HD Anié	113 (27,2)	10(8,8)	6,47 [1,74-24,03]	0,005
Kouvé	99 (23,9)	10(10,1)	7,49 [2,01-27,88]	0,003
Sexe				
féminin	185 (44,6)	12(6,5)	1	
masculin	230 (55,4)	11(4,8)	0,72 [0,31-1,68]	0,452
Âge en années				
<5	212 (51,1)	20(9,4)	1	
[5-34]	55 (13,3)	3(5,5)	0,55 [0,16-1,94]	0,355
≥35	148 (35,7)	0(0)	1	1
Température				
<37,5	92 (22,2)	6(6,5)	1	
≥37,5	323 (77,8)	17(5,3)	0,80 [0,30-2,08]	0,642
Automédication				
non	336 (81,0)	22(6,5)	1	
oui	79 (19,0)	1(1,3)	0,18 [0,02-1,38]	0,099
Profession				
travail salarié	83 (20,0)	0(0)	1	
travail à la tâche	96 (23,1)	1(1,1)	0,13 [0,02-1,00]	0,051
élève/étudiant	69 (16,6)	10(14,5)	2,05 [0,84-4,99]	0,115
enfant	157 (37,8)	12(7,6)	1	1
autre	10 (2,4)	0(0)	1	1
Résidence				
urbain	268 (64,6)	11(4,1)	1	
rural	147 (35,4)	12(8,2)	2,08 [0,89-4,83]	0,09

## Discussion

Cette étude présente un certain nombre de limites. Le recrutement des participants dans notre étude ayant été réalisé dans les formations sanitaires situées toutes au sud du pays, nos résultats ne peuvent donc pas être extrapolés à tout le pays. Par ailleurs, la population considérée était celle des patients symptomatiques, l’évaluation de l’IS n'a donc pas inclus les infections asymptomatiques. En dépit de ces limites, notre étude a mis en lumière l'existence des cas d’IS au Togo qu'il faudra prendre en compte dans les stratégies futures de la lutte antipaludique. Nos résultats par la microscopie étaient issus d'un contrôle en double lecture par deux microscopistes expérimentés dont l'un est accrédité par l’OMS niveau 2 pour le diagnostic du paludisme. Habituellement, autant d'attention n'est pas accordée pour la lecture en routine des lames de GE/FS du fait de la charge de travail du personnel technique dans les laboratoires de pays à ressources limitées.

Davantage de cas à parasitémie faible échappent à la détection des cas positifs par la microscopie considérée jusque-là comme la méthode de référence en pratique routinière.

Nous n'avons pas utilisé de TDR. Outre leur sensibilité similaire à celle de la microscopie, ils ne détectent pas des parasites mais des antigènes présents dans le sang des personnes infectées, que l'infection soit récente ou non. Ils peuvent donc constituer de faux positifs. En outre, il n'entrait pas dans notre objectif de valider cette technique dans le cadre de la détection d'infections submicroscopiques. Cependant, leur utilisation en routine permet un résultat similaire avec l'avantage de ne pas nécessiter un personnel qualifié en microscopie.

Dans notre étude, le portage submicroscopique a été influencé par la période à laquelle les patients ont été enrôlés : les inclus des sites d’Anié et de Kouvé reçus de septembre à janvier, avaient respectivement 6 et 7 fois plus de risque de présenter une infection submicroscopique que ceux du site de CHR-LC reçus de juillet à décembre, période ayant un lien étroit avec la modulation de la transmission palustre dans le pays. L’évolution des cas de paludisme dans le sud du pays (régions Grand Lomé, Maritime et Plateaux) est marquée par deux pics de mai à juillet et de septembre à novembre, périodes correspondant à la saison des pluies caractérisées par un fort taux d'inoculation et de transmission palustre. Nos données sont corroborées par les résultats de Whittaker *et al.* [30] qui ont démontré lors d'une revue systématique et d'une méta-analyse, l'influence significative des saisons sur les IS, qui sont plus fréquentes pendant la saison sèche. Cette variation pourrait se justifier par le fait que pendant les saisons des pluies, les densités parasitaires augmentent légèrement [24], devenant plus susceptibles d’être détectées par la microscopie. Ainsi, les sites de notre étude étant tous localisés dans le sud du Togo, la variation notée entre les sites d’Anié et de Kouvé et celui du CHR-LC serait liée à la période d'enrôlement, les patients du CHR-LC étant enrôlés de septembre à janvier, période caractérisée par une relative faible pluviométrie. Cette différence peut également être liée au fait que les deux premiers sites étaient des hôpitaux de district, représentant le premier niveau de contact avec les patients alors que le CHR-LC, structure de niveau intermédiaire, était un centre de référence, les patients qui y étaient admis avaient peut-être été déjà traités pour le paludisme avant leur admission. En outre, ce dernier site draine majoritairement une population urbaine probablement sujette à une transmission moindre que celle des deux autres zones.

Dans cette étude, l’âge n’était pas associé à un portage élevé de parasites. La fréquence des IS était plus élevée chez les moins de cinq ans. Nos résultats contrastent avec ceux de la littérature puisqu'il a été prouvé que les adultes ont des niveaux d'immunité plus élevés et maintiennent les parasites à de faibles densités [8, 22, 25] et qu'en zones d'endémie palustre, le développement d'une immunité adaptative due à une exposition fréquente au *Plasmodium* spp., est fonction de l’âge [3]. De ce fait, les adultes sont davantage porteurs de parasites asymptomatiques comparés aux enfants dont l'immunité antipaludique est en développement [33]. Aucune différence n'a été non plus notée entre l’IS et la présence de fièvre dans notre étude. Cette fièvre, un des premiers symptômes de l'accès palustre, est présente chez les sujets ayant un résultat positif à la PCR. Elle aurait pu davantage conforter notre diagnostic d’IS bien qu'un portage plasmodial asymptomatique et une autre pathologie fébrile puissent coexister chez le même patient [31]. C'est le cas pour d'autres maladies vectorielles fébriles comme la dengue, le Zika et le chikungunya qui ont la fièvre en symptôme commun avec le paludisme [10].

Malgré ces considérations, la PCR semble être le meilleur outil diagnostique (c'est-à-dire le plus sensible) pour l'estimation de la prévalence du parasite dans la population générale. Une étude complémentaire sur cette IS, qui prendrait en compte non seulement les patients asymptomatiques mais couvrirait également la partie nord du pays dont le profil épidémiologique palustre est différent, aiderait à confirmer nos résultats préliminaires. Par ailleurs, des études longitudinales avec davantage de variables telles que l'utilisation de moustiquaires imprégnées, la prise récente de déparasitant et le nombre de personnes vivant dans le ménage pourraient enrichir l'analyse des facteurs associées à l’IS. En vue de limiter le risque de survenue de cas d’IS, nos résultats doivent inciter les autorités sanitaires à mettre en œuvre des formations continues pour la mise à niveau du personnel de laboratoire, en insistant davantage sur le respect strict du temps de lecture et de l'examen du nombre minimal requis de champs microscopiques en pratique de routine de confirmation parasitologique du paludisme [18]. Cette étude suggère également de compléter la microscopie par des tests de diagnostic rapide du paludisme ultrasensibles (à défaut de la PCR) ciblant les patients présentant des symptômes similaires à ceux du paludisme.

## Conclusion

Une faible proportion d’IS à *Plasmodium* spp. a été retrouvée dans les trois sites couverts par notre étude au Togo. Ces individus porteurs de parasites non détectés par les examens de routine, constituent un réservoir pouvant entretenir la transmission du paludisme dans la population. La mise en place d'approches pour améliorer leur détection et envisager leur prise en charge thérapeutique par les nouvelles stratégies adaptées s'avère nécessaire pour accélérer l'atteinte de l'objectif mondial d’élimination du paludisme dans le monde.

## Remerciements et sources de financement

Cette étude a bénéficié du soutien de la Division des laboratoires, du Programme national de lutte contre le paludisme du Togo, de l’Université de Lomé et de l’Université de Strasbourg. L'analyse PCR des confettis a été rendue possible grâce au soutien financier du Fonds mondial de lutte contre le VIH/SIDA, la tuberculose et le paludisme au niveau national et de la Fondation Bill et Melinda Gates par l'intermédiaire de l’OMS-Genève. Les auteurs remercient les directeurs régionaux et préfectoraux de la santé des trois sites d’étude, l’équipe de recherche et les membres des équipes locales des sites pour leur implication à tous les niveaux. Les auteurs remercient également les participants à l’étude ainsi que leurs parents et tuteurs des enfants.

## Contribution des auteurs et autrices

Teou DC, Dorkenoo AM, Ataba E et Yakpa K ont rédigé le protocole d’étude.

Dorkenoo AM a dirigé la formation et supervisé la mise en œuvre du projet.

Dorkenoo AM, Ataba E, Yakpa K, Sossou E, Hemou M, Abdou-Kerim A, Kotosso A et Bawe LD ont supervisé la collecte des données. Ménard D a supervisé l'analyse moléculaire et la validation desdits résultats.

Teou DC et Alidou S ont analysé et interprété les résultats.

Teou D.C, Dorkenoo A.M, Ataba E, Ménard D et Alidou S ont rédigé la première version du manuscrit.

Tous les auteurs ont lu, révisé et approuvé le manuscrit final.

## Conflit d'intérêt

Les auteurs ne rapportent aucun conflit d'intérêt.
