# Supplementary material for: Submicroscopic Plasmodium spp. Infections in Febrile Patients in Togo
Source: Med Trop Sante Int. 2025 Feb 27;5(1):mtsi.v5i1.2025.553. [Article in French] doi: 10.48327/mtsi.v5i1.2025.553 (PMC12001993; doi:10.48327/mtsi.v5i1.2025.553)
Supplement: Related texts

# Research article: Submicroscopic *Plasmodium* spp. Infections in Febrile Patients in Togo

## Introduction

The ability of health systems in malaria-endemic countries to detect *Plasmodium* spp. infections is essential for effective surveillance and control of the endemic disease [30]. The World Health Organization (WHO) [19] recommends the use of artemisinin-based combination therapies (ACTs) for the treatment of uncomplicated *P. falciparum* cases and intravenous or intramuscular artesunate for at least 24 hours, supplemented by ACTs for severe malaria cases.

In Togo, in addition to these recommendations, which have been implemented since 2005 [11], other prevention strategies have been successfully implemented, including the distribution of long-lasting insecticide-treated nets, intermittent preventive treatment with sulfadoxine-pyrimeth-amine in pregnant women and seasonal malaria chemoprevention in children aged 3-59 months with the combination of sulfadoxine-pyrimeth-amine and amodiaquine during the high transmission season, as well as improved diagnosis and treatment of confirmed cases. They could explain the observed decline in malaria prevalence in children aged 6-59 months from 36% to 28% between 2014 and 2017 [15]. In terms of deaths, the malaria case fatality rate was 2.1% in 2022, compared with 3.6% in 2019 [16].

National guidelines recommend that all suspected cases of malaria be confirmed by a thick blood film (TBF), blood smear (BS) or rapid diagnostic test (RDT) before any antimalarial treatment. Because of their ease of use and rapid availability of results, RDTs were first used in health facilities without microscopy and by community health workers, and more recently in health facilities with microscopy. The RDTs currently in use are specific for *P. falciparum* species [16]. However, TBF/BS microscopic examination remains the benchmark; it allows the detection of different stages of *Plasmodium* species and the estimation of parasite density (PD). The detection threshold for *Plasmodium* species using this approach, estimated to be between 10 and 20 parasites/µL [9], remains much higher than that of molecular biology (polymerase chain reaction, PCR), which is estimated to be 0.002 parasites/µL [29], making it possible to detect cases of submicroscopic infection (SI). In resource-limited and malaria-endemic countries, PCR is often used for biological confirmation during epidemiological studies and evaluation of antimalarial drug efficacy, but not routinely [9, 12].

The proportion of IS varies according to the epidemiological context and the populations affected [21, 27]. The contribution of this reservoir to sustaining malaria transmission depends on the endemicity and the positivity rate of malaria cases detected in a given area [12].

Okell *et al.* reported that, globally, the prevalence of IS is inversely correlated with PD [17] .Other studies have also suggested that the proportion of SI is often higher than microscopically detectable in low transmission areas and is likely to increase as control programs continue to reduce the intensity of malaria transmission [5, 13] .It has also been shown that the incidence of SI is not negligible in high transmission areas and that these infections play a role in the development of partial, non-sterilizing immunity to malaria [26]. In West Africa, some studies have documented cases of SI. In Benin, Agohossou *et al.* [2] and Telfils *et al.* [28] reported a prevalence of submicroscopic infections of 30.6% and 30.4%, respectively. In Ghana, Amoah *et al.* [4] reported a prevalence of 33.8%. In Togo, submicroscopic infections have not yet been documented. This study was conducted to estimate the proportion of submicroscopic *Plasmodium* spp. infections not detected by microscopy in symptomatic malaria suspects attending health centers in Togo and to identify factors associated with them.

## Materials and methods

A cross-sectional study was conducted between September 2021 and January 2022 at the ‘La Providence’ hospital in Kouvé and the District Hospital (DH) in Anié, and between July and December 2022 at the Lomé Commune Regional Hospital Centre (CHR-LC). The La Providence hospital in Kouvé, located in the maritime region, is about 76 km from Lomé, and the Anié district hospital, located in the plateau region, is 188 km from Lomé. These two health facilities are among the six sentinel sites established by the Ministry of Health to monitor the effectiveness of antimalarial drugs. They are the first point of contact with an health facility for the patients. To take account of the second level of the pyramidal organization of the Togolese health system, the CHR-Lomé commune, an intermediate structure located in the Greater Lomé Health Region, has been added. It covers the geographical area of the capital Lomé and its suburbs. These three sites represent the three health regions in the south of the country.

The study population consisted of symptomatic patients with suspected malaria for whom TBF/ BS tests were prescribed. Any patient, regardless of age, who presented to the general outpatient clinic of one of the participating health facilities with an axillary temperature > 37.5°C (or a history of fever in the previous 24 hours with suspected malaria) was included in the study. They had to have given informed consent (directly or, in the case of children, through their parents/guardians). After completing the form used to collect socio-demographic information, presenting clinical symptoms and medical history, each selected patient had a capillary blood sample taken for TBF/ BS and blood confetti on Wattman type III paper. TBF and BS, prepared on the same slide, were stained with 3% Giemsa after the BS was fixed in methanol for a few seconds [20]. After drying, the slides were examined under a microscope at x100 magnification to determine positivity, identify the *Plasmodium* species and estimate the PD. This PD was expressed as the number of asexual plasmodia per microliter of blood according to the formula: number of plasmodia counted x 8,000 / number of leukocytes counted.

For DNA extraction, blood confetti were cut out with scissors and transferred to 96-well plates. Genomic DNA was extracted using the Qiagen RLT-plus® Kit according to the manufacturer's protocol with some modifications [1]. Each well was filled with 750 µL lysis buffer, 500 µL wash buffer 1, 500 µL wash buffer 2 and 100 µL elution buffer (Tris-EDTA). Extraction was performed at the recommended temperature, incubation time and centrifugation speed as indicated by the manufacturer. Finally, the elution plates containing the extracted DNA were stored at -80°C until use. Samples were screened for the presence of *Plasmodium* spp. DNA using a qualitative real-time PCR assay targeting the cytochrome b gene. The products for the positive samples were then diluted 1:10 and analyzed using a real-time PCR assay with primers targeting the same gene fragment and specific for each species (PCR for the species *P. falciparum, P. vivax*, *P. malariae* and *P. ovale).* All real-time assays were performed using the master mix PCR SYBR green (Solis Biodyne, Tartu, Estonia) on a 5 µL DNA extract using the BioRad CFX96 real-time PCR system. Details of the program, mixture composition and primer sequences have been described by Canier *et al.* [6, 7].

The TBF/BS slides of the included subjects were reviewed by two experienced microscopists [18]. In cases where the coefficient of variation between the results of the two quality control microsco-pists was greater than 5%, a third reading was performed by another independent microscopist [20]. In the event of a difference between the PD observed in the health facilities and the one by the quality control team we considered the quality control PD.

For each PCR screening analysis, a set of four controls (two positive controls, one high positive and one low positive, and two negative controls corresponding to water) was added to each 96-well plate. To validate the species, the plasmid constructs described above were used in each PCR assay [7].

Training was provided to the field team members and supervisors to standardize working methods and ensure the smooth running of the activity, particularly for the completion of questionnaires, the performance of the TBF/BS and the collection of samples on filter paper.

The dependent variable is a binary variable indicating the presence (positive: coded 1) or absence (negative: coded 0) of submicroscopic infection. This infection is defined as a low density parasitic infection in the blood stage of malaria not detected by TBF/BS, but only by PCR, which is considered the reference method [18].

The independent variables include the study sites, namely CHR-LC, HD d’Anié and Kouvé hospital, as well as socio-demographic and clinical characteristics. The sex of the participants is distinguished (female or male) and the age is divided into three groups: under 5 years, 5 to 34 years and 35 years and older. Temperature was categorized as normal <37.5°C or high ≥37.5°C, and self-medication was coded as absent (no) or present (yes). For occupation, participants were categorized as employees, pieceworkers, students, children, and others. Finally, residence distinguished between urban and rural residents. These variables were selected based on their relevance in the existing literature and have been analyzed in similar studies [12, 14, 23, 32].

All data collected were entered into a database in Microsoft Excel (Microsoft Corp, Redmond, Washington, USA) and analyzed using Stata 16 (StataCorp LLC). Qualitative data were presented as proportions followed by their 95% CIs, and quantitative data were described as medians with their interquartile ranges. Samples that were negative for both TBF/BS and PCR were compared with those that were negative for TBF/BS and positive for PCR. For these analyses, Fisher's exact test was used to compare groups, and logistic regression was used to identify a possible association with the factors studied. A p-value < 0.05 was considered statistically significant. Variables with a p-value of less than or equal to 0.20 in the preliminary analysis were considered for multiple logistic regression.

The study protocol was approved by the Togolese Bioethics Committee for Health Research (CBRS) (No. 021/2021/CBRS of May 24, 2022). In addition, a signed informed consent form was required from adults or parents/guardians in the case of children. Any patient testing positive for TBF/ BS was referred to clinicians at participating facilities for free treatment with artemether-lumefantrine or dihydroartemisinin-piperaquine as recommended by the National Malaria Control Program (NMCP).

## Results

A total of 553 patients with suspected malaria were included across all sites: 138 had a positive TBF (Table I). The search for cases of submicroscopic infections by PCR screening was carried out on the 415 negative samples by TBF/BS (Fig. 1).

**Table I:** Total number of samples included by site

Sites	Kouvé n (%)	HD Anié n (%)	CHR-LC n (%)
TBF/BS			
**negative (n = 415)**	**99 (17,9)**	**113 (20,4)**	203 (36,7)
**positive (n = 138)**	**35 (6,3)**	**103 (18,6)**	0 (0)
**total (n = 553)**	**134 (24,2)**	**216 (39,1)**	203 (36,7)
PCR			
**negative (n = 392)**	**89 (16,1)**	**103 (18,6)**	200 (36,2)
**positive (n = 161)**	**45 (8,1)**	**113 (20,4)**	3 (0,5)
**total (n = 553)**	**134 (24,2)**	**216 (39,1)**	203 (36,7)

**Figure 1:** Study flow chart

The average age of the patients was 25 years (±2 years) and the sex ratio (M/F) was 1.2. A body temperature on admission of ≥ 37.5°C was recorded in 77.8% of them, the others having had a history of fever in the 24 hours prior to the consultation. The socio-demographic data of the patients are summarized in Table III.

The detection of the *Plasmodium* genome by PCR was carried out on all 553 included (Table I). Comparing the two methods, PCR detected more positive cases, i.e. 29.1% (161/553) against 25% (138/553) by microscopy (Table I).

The frequency of submicroscopic *Plasmodium* spp. infections was 5.5% (23/415) [95% CI: 3.7-8.2]. The majority of these infections were by *P. falciparum*, i.e. 83% (19/23) [95% CI: 60-94] of cases. *P. malariae* was found in 9% (2/23) [95% CI: 2-31] of these submicroscopic infections, as was the *P. falciparum-P. malariae* association, in 9% (2/23) [95% CI: 2-31] of cases (Table II). No cases of submicroscopic *P. vivax* or *P. ovale* infection were identified among the patients studied. Among the 415 samples that were TBF/BS-negative and tested by PCR, the participants from the Anié and Kouvé sites had 6 (OR, 6.47 [95% CI: 1.74-24.03]; p = 0.005) and 7 (OR, 7.49 [95% CI: 2.01-27.88]; p = 0.003) times more likely to have an SI. Although the frequency of these infections was higher in children under five years of age, no significant difference based on age or sex was observed. Self-medication, occupation and place of residence were also not associated with submicroscopic infection (Table III).

**Table II:** Frequency of submicroscopic parasitemia and identified *Plasmodium* species

Sites	Kouvé	HD Anié	CHR-LC	Total [IC 95 %]
TBF/BS-negative (n)	99	113	203	415
subicroscopy n (%)	10 (10,1)	10 (8,8)	3 (1,5)	23 (5,5) [03,7-08,2]
*Plasmodium* species				
Pf*, n (%)	9	7	3	19
Pm*, n (%)	1	1	0	2
*Pf/Pm,* n (%)	0	2	0	2

* *Pf : Plasmodium falciparum, Pm : Plasmodium malariae*

**Table III:** Association between demographic characteristics and sub-microscopic *Plasmodium* spp. infections

Characteristics	Frequency n (%)	Submicroscopic infections n (%)	Odds ratio (IC 95 %)	p-value
Study site				
CHR-LC	203 (48,9)	3(1,5)	1	
HD Anié	113 (27,2)	10(8,8)	6,47 [1,74-24,03]	0,005
Kouvé	99 (23,9)	10(10,1)	7,49 [2,01-27,88]	0,003
Gender				
female	185 (44,6)	12(6,5)	1	
male	230 (55,4)	11(4,8)	0,72 [0,31-1,68]	0,452
Age in years				
<5	212 (51,1)	20(9,4)	1	
[5-34]	55 (13,3)	3(5,5)	0,55 [0,16-1,94]	0,355
≥35	148 (35,7)	0(0)	1	1
Temperature (°C)				
<37,5	92 (22,2)	6(6,5)	1	
≥37,5	323 (77,8)	17(5,3)	0,80 [0,30-2,08]	0,642
Self-medication				
no	336 (81,0)	22(6,5)	1	
yes	79 (19,0)	1(1,3)	0,18 [0,02-1,38]	0,099
Occupation				
salaried work	83 (20,0)	0(0)	1	
piecework	96 (23,1)	1(1,1)	0,13 [0,02-1,00]	0,051
student	69 (16,6)	10(14,5)	2,05 [0,84-4,99]	0,115
child	157 (37,8)	12(7,6)	1	1
other	10 (2,4)	0(0)	1	1
Residence				
urban	268 (64,6)	11(4,1)	1	
rural	147 (35,4)	12(8,2)	2,08 [0,89-4,83]	0,09

## Discussion

This study has several limitations. Because the participants in our study were recruited from health facilities all located in the south of the country, our results cannot be extrapolated to the whole country. In addition, the population considered was that of symptomatic patients, so the assessment of SI did not include asymptomatic infections. Despite these limitations, our study has highlighted the existence of SI cases in Togo, which must be taken into account in future malaria control strategies. Our microscopy results were derived from a double reading control by two experienced microscopists, one of whom is WHO level 2 accredited for malaria diagnosis. Routine reading of TBF/BS slides is not usually given such close attention due to the workload of technical staff in laboratories in resource-limited countries. It is conceivable that more cases with low parasitemia escape detection as positive cases by microscopy, which has been considered the reference method in routine practice.

We did not use RDTs. In addition to their sensitivity, which is similar to that of microscopy, they do not detect parasites, but antigens present in the blood of infected individuals, whether the infection is recent or not. They can therefore produce false positives. Furthermore, it was not our aim to validate this technique in the context of detecting submicroscopic infections. However, its routine use provides a similar result, with the advantage of not requiring personnel trained in microscopy.

In our study, submicroscopic carriage was influenced by the period in which the patients were enrolled: the samples from the Anié and Kouvé sites received from September to January had a 6 and 7 times higher risk of presenting a submicroscopic infection, respectively, than those from the CHR-LC site received from July to December, a period closely linked to the modulation of malaria transmission in the country. The evolution of malaria cases in the south of the country (Grand Lomé, Maritime and Plateaux regions) is characterized by two peaks from May to July and from September to November, periods corresponding to the rainy season, characterized by a high rate of malaria transmission. Our data are corroborated by the results of Whittaker *et al.* [30] who, in a systematic review and meta-analysis, demonstrated the significant influence of the seasons on SI, more frequent during the dry season. This variation could be explained by the fact that during the rainy season, parasite densities increase slightly [24] and are more likely to be detected by microscopy. As the sites in our study are all located in the south of Togo, the variation observed between the Anié and Kouvé sites and the CHR-LC site would be related to the enrollment period, with CHR-LC patients enrolled from September to January, a period characterized by relatively low rainfall. This difference may also be related to the fact that the first two sites were district hospitals, representing the first level of contact with patients, while the CHR-LC, an intermediate level structure, was a reference center. Patients admitted there may have already been treated for malaria prior to admission. In addition, the latter site mainly serves an urban population, which is likely to be less exposed to transmission than the other two areas.

In this study, age was not associated with a high parasite burden. The frequency of SI was higher in children under five years of age. Our results contrast with those in the literature. It has been shown that adults have higher levels of immunity and maintain parasites at low densities [8, 22, 25]. In areas where malaria is endemic, the development of adaptive immunity due to frequent exposure to *Plasmodium* spp. depends on age [3]. As a result, adults are more likely to be asymptomatic parasite carriers than children, whose antimalarial immunity is still developing [33]. In our study, no difference was found between SI and the presence of fever. This fever, one of the first symptoms of malaria, is present in subjects with positive PCR results. It could have further supported our diagnosis of SI, although asymptomatic *Plasmodium* carriage and another febrile pathology may coexist in the same patient [31]. This is the case for other febrile vector-borne diseases such as dengue, Zika, and chikungunya, which share fever as a symptom with malaria [10]. Despite these considerations, PCR seems to be the best diagnostic tool (i.e. the most sensitive) for estimating the prevalence of the parasite in the general population. A complementary study on this SI, which would not only include asymptomatic patients but also cover the northern part of the country, where the epidemiologic profile of malaria is different, would help to confirm our preliminary results. In addition, longitudinal studies with more variables such as the use of impregnated bed nets, recent antimalarial treatments, and the number of people living in the household could enrich the analysis of factors associated with SI. In order to limit the risk of SI cases, our results should encourage health authorities to implement continuous training of laboratory staff, with greater emphasis on strict adherence to reading time and examination of the minimum number of microscopic fields required for routine parasitological confirmation of malaria [18]. This study also suggests complementing microscopy with ultrasensitive rapid diagnostic tests for malaria (in the absence of PCR), targeting patients with symptoms like those of malaria.

## Conclusion

A low proportion of *Plasmodium* spp. IS was found in the three sites included in our study in Togo. These individuals carrying parasites not detected by routine examinations represent a reservoir that can maintain malaria transmission in the population. Approaches to improve their detection and consider their therapeutic management through new and adapted strategies are necessary to accelerate the achievement of the global goal of eliminating malaria worldwide.

## Acknowledgements and funding

This study was supported by the Laboratory of the National Malaria Control Program of Togo, the University of Lomé and the University of Strasbourg. The PCR analysis of confetti was made possible thanks to the financial support of the Global Fund to Fight HIV/AIDS, Tuberculosis and Malaria at the national level and the Bill & Melinda Gates Foundation through WHO Geneva. The authors would like to thank the regional and prefectural directors of health in the three study sites, the research team, and the members of the local site teams for their commitment at all levels. The authors would also like to thank the study participants and their parents and guardians.

## Authors’ contributions

Teou DC, Dorkenoo AM, Ataba E, and Yakpa K drafted the study protocol.

Dorkenoo AM led the training and supervised the implementation of the project.

Dorkenoo AM, Ataba E, Yakpa K, Sossou E, Hemou M, Abdou-Kerim A, Kotosso A, and Bawe LD supervised the data collection.

Ménard D supervised the molecular analysis and validation of the results.

Teou DC and Alidou S analyzed and interpreted the results.

Teou D.C, Dorkenoo A.M, Ataba E, Ménard D and Alidou S drafted the first version of the manuscript.

All authors read, revised, and approved the final manuscript.

## Conflicts of interest

The authors declare no conflict of interests.
